# Associations Between Chemotherapy-Induced Peripheral Neuropathy and Distress in Patients Assigned to Adjuvant Irradiation for Non-Metastatic Breast Cancer

**DOI:** 10.3390/jpm15060248

**Published:** 2025-06-13

**Authors:** Dirk Rades, Tobias Bartscht, Achim Rody, Martin Ballegaard

**Affiliations:** 1 Department of Radiation Oncology, University of Lübeck, 23562 Lübeck, Germany; 2 Department of Radiation Oncology, University Medical Center Schleswig-Holstein, Lübeck Campus, 23538 Lübeck, Germany; 3 Department for Human Medicine, MSH Medical School Hamburg, 20457 Hamburg, Germany; tobias.bartscht@helios-gesundheit.de; 4 Department of Hematology, Oncology and Stem Cell Transplantation, Helios Hospital Schwerin, 19055 Schwerin, Germany; 5 Department of Obstetrics and Gynecology, University Medical Center Schleswig-Holstein, Lübeck Campus, 23538 Lübeck, Germany; achim.rody@uksh.de; 6 Department of Neurology, Zealand University Hospital, 4000 Roskilde, Denmark; mbag@regionsjaelland.dk; 7 Department of Clinical Medicine, Faculty of Health and Medical Sciences, University of Copenhagen, 2200 Copenhagen, Denmark

**Keywords:** breast cancer, paclitaxel, docetaxel, peripheral neuropathy, distress, adjuvant radiotherapy

## Abstract

**Background/Objectives:** Many patients assigned to adjuvant radiotherapy for non-metastatic breast cancer already received taxane-based chemotherapy, which can cause peripheral neuropathy (PNP). This study investigated potential associations between moderate-to-severe or mild PNP and distress. **Methods:** Ninety-eight breast cancer patients who received taxane-based chemotherapy and completed the National Comprehensive Cancer Network Distress Thermometer were included in this retrospective study. The severity of PNP plus 17 factors were evaluated for associations with distress. **Results:** Mean distress scores (higher scores representing higher levels of distress) were 6.17 (SD ± 2.41) in patients with moderate-to-severe PNP, 4.21 (SD ± 2.54) in patients with mild PNP and 4.04 (SD ± 2.24) in patients without PNP. On univariable analyses, higher distress scores were significantly associated with moderate-to-severe PNP (vs. mild or no PNP, *p* < 0.001), Karnofsky performance score ≤ 80 (*p* = 0.001), history of autoimmune disease (*p* = 0.035), and hypertension (*p* = 0.002). Trends were found for age ≥65 years (*p* = 0.056), type of chemotherapy (*p* = 0.078), and beta-blocker medication (*p* = 0.072). On multivariable analysis, moderate-to-severe PNP (*p* = 0.036), Karnofsky performance score ≤ 80 (*p* = 0.013), and hypertension (*p* = 0.045) were significant. **Conclusions**: Since moderate-to-severe chemotherapy-induced PNP was associated with a significantly higher level of distress when compared to mild or nor PNP, these patients should be offered early psychological support and personalized monitoring.

## 1. Introduction

The treatment of non-metastatic breast cancer includes surgery, i.e., breast-conserving surgery or mastectomy [1,2,3]. After breast-conserving surgery, patients need adjuvant radiotherapy. Radiation treatment may also be required following mastectomy, depending on the primary tumor and nodal stage and other risk factors. The presence of risk factors could require additional treatment with pre- or postoperative chemotherapy prior to the start of the radiotherapy course. The corresponding chemotherapy regimens are generally taxane-based. Taxanes, namely paclitaxel and docetaxel, are associated with a significant risk of peripheral sensory neuropathy (PNP) [4]. Symptoms of PNP, which can be classified as mild or moderate to severe, can include numbness, pain, and tingling, mostly affecting hands and feet [5]. The situation can be quite burdensome for the patients, including an increased risk of falling [6,7,8]. Following the course of chemotherapy, PNP may improve or persist as a long-term complication [8,9,10,11].

Several authors reported that chemotherapy-induced PNP can negatively affect the quality of life of patients with breast cancer [12,13,14,15,16,17,18,19]. However, there is a lack of studies focusing on associations between PNP and (psychological) distress, as concluded by the authors of a scoping review published in 2023 [20]. They identified only eight studies that addressed symptoms of psychological distress in breast cancer patients with chemotherapy-induced PNP [20].

When looking at the corresponding articles in more detail, it becomes obvious that the investigated endpoints were quite heterogeneous. Endpoints included the impact of post-operative chemotherapy on PNP in general (versus surgery alone), impact of the type of chemotherapy, impact of pre-treatment anxiety and depression, pain profiles and cause of pain, impact of persisting docetaxel-induced PNP on quality of life, impact of physiologic, psychological and situational factors on symptoms of PNP, impact of symptom experience on functional interference regarding daily activities, incidence/prevalence of PNP, risk factors of PNP, and their impact on patient-reported outcomes or psychological distress and on fall risk [8,9,11,21,22,23,24,25]. In addition, a retrospective study investigated between the onset of chemotherapy-induced PNP and the development of anxiety and depression in breast cancer survivors [26]. No study so far has investigated the potential impact of chemotherapy-induced PNP on the level of distress in breast cancer patients using the National Comprehensive Cancer Network (NCCN) Distress Thermometer [27,28,29]. This study was performed to close this gap. The main goal of this study was to identify associations between mild or moderate-to-severe chemotherapy-induced PNP and distress in a cohort of patients assigned to adjuvant radiotherapy for non-metastatic breast cancer. If such associations can be demonstrated, the results of our study will likely increase the awareness of radiation oncologists with respect to chemotherapy-induced PNP and the initiation of supporting measures such as routine physical therapy, vibration therapy, and photo-biomodulation, although treatment options for PNP are generally limited [30,31,32,33,34,35,36]. Moreover, the patients would likely benefit from early psychological support and personalized monitoring during and following their treatment course. The current study is part of the German-Danish Interreg-project Health Advancing Technologies for Elderly (HeAT).

## 2. Materials and Methods

Ninety-eight female breast cancer patients who received taxane-based chemotherapy and completed the German version of the NCCN Distress Thermometer [27,28,29] were included in this retrospective study, which received approval from the responsible Ethics Committee at the University of Lübeck, Germany (file 2025-100). The NCCN Distress Thermometer comprises two parts. In the first part, the patients indicate how much distress they have been experiencing during the past week (including today). Distress scores range between 0 (no distress) and 10 (maximum distress). The second part includes a list of problems in different areas (practical problems, family problems, emotional problems, spiritual/religious concerns, physical problems). The patients are asked to indicate if any of the items have been a problem for them during the past week (including today).

The patients were assigned to adjuvant radiotherapy for non-metastatic breast cancer in 2022 or 2023. In total, 97 of 98 patients were Non-Hispanic Whites. For many patients, information regarding their education, socio-economic status, and employment was not available. Upfront surgery related to breast cancer was breast-conserving surgery in 75 patients and mastectomy in 23 patients. Taxane-based chemotherapy was administered pre-operatively (neoadjuvant) in 59 patients and post-operatively (adjuvant) in 39 patients. Moreover, 77 patients received chemotherapy including paclitaxel and 21 patients including docetaxel. The chemotherapy regimens were (a) epirubicin/cyclophosphamide + paclitaxel (EC + PAC), (b) epirubicin/cyclophosphamide + paclitaxel/carboplatin (EC + PAC/Carbo), (c) epirubicin/cyclophosphamide + paclitaxel/carboplatin + pembrolizumab (EC + PAC/Carbo + Pembro), (d) paclitaxel + trastuzumab (APT), (e) epirubicin/paclitaxel/cyclophosphamide (ETC), and (f) docetaxel/carboplatin/trastuzumab/pertuzumab (TCbHP) (Table 1).

For radiotherapy of the whole breast or the chest wall, high-precision techniques were used, namely intensity-modulated radiation therapy (IMRT) in 54 patients and volumetric modulated arc therapy (VMAT) in 44 patients. Ninety-four patients had unilateral (50 right breast, 44 left breast) and four patients bilateral breast cancer. In patients with unilateral involvement, radiotherapy was administered to the breast alone in 47 patients, to the breast and regional lymph nodes in 27 patients, to the chest wall alone in 2 patients, and to the chest wall and lymph nodes in 18 patients, respectively. In total, 51 of the 94 patients with unilateral involvement received hypo-fractionated irradiation with 40 Gy in 15 fractions, and 43 patients normo-fractionated irradiation with 50.4 Gy in 28 fractions.

In total, 73 of the 94 patients received a radiation boost to the former tumor bed, which was administered sequentially with 10 Gy in five fractions in 65 patients and simultaneously as an integrated boost of 0.3 Gy per radiation fraction, respectively.

Of the four patients with bilateral cancer, two patients required only unilateral irradiation without a boost. In one patient, radiotherapy was administered to the chest wall alone (40 Gy in 15 fractions). In the other patients, the treatment volume included the chest wall and the regional lymph nodes (50.4 Gy in 28 fractions). The other two patients received normo-fractionated irradiation, which was administered to the breast alone on one side and to the chest wall and lymph nodes on the other side. One of these patients received a simultaneous integrated boost to the former tumor bed in addition to radiotherapy to the breast alone.

In the entire cohort, 35 patients experienced mild sensory PNP and 26 patients moderate-to-severe sensory PNP, respectively. In addition to sensory PNP, one and seven of these patients, respectively, appeared to have motor symptoms. Seventeen additional characteristics (Table 2) were evaluated for potential associations with distress. PNP was categorized as mild (mild symptoms like tingling or numbness with minimal impact on daily activities) or moderate (more intense symptoms that limit instrumental activities of daily living) to severe (significant symptoms that limit self-care activities of daily living) [37]. The assessment of PNP was performed by the treating gynecologists and radiation oncologists, and the diagnosis of PNP was based on patient-reported symptoms and physical examination. With respect to PNP, two analyses were performed. One analysis included three categories (moderate-to-severe PNP vs. mild PNP vs. no PNP) and the other analysis two categories (moderate-to-severe PNP vs. mild or no PNP). The additional characteristics included the time of chemotherapy in relation to surgery (neoadjuvant vs. adjuvant), type of surgery (breast-conserving surgery vs. mastectomy), age (≤64 vs. ≥65 years), Karnofsky performance score (≤80 vs. 90–100), body mass index (BMI <25.0 kg/m^2^ vs. 25.0–29.9 kg/m^2^ vs. ≥30.0 kg/m^2^), personal status (living with spouse or partner vs. single/widow/living alone), other malignancy (no vs. yes), type of chemotherapy [EC + PAC vs. EC + PAC/Carbo vs. EC + PAC/Carbo + Pembro vs. APT vs. ETC vs. TCbHP (Abbreviations used for the types of chemotherapy are explained in Table 1.)], autoimmune disease (no vs. yes), significant cardiovascular disease (no vs. yes), hypertension (no vs. yes), diabetes (no vs. yes), history of smoking (<10 vs. ≥10 pack years), beta-blocker medication (no vs. yes), main histology (no special type alone vs. other), histologic grading (G1 or G2 vs. G3), and triple negativity (no vs. yes). Primary tumor and nodal stage were not considered for the analyses with respect to distress, because they were not uniform (clinical, pathological, or post-chemotherapy) due to the fact that this study included both patients with neoadjuvant and adjuvant chemotherapy. Patients with other malignancies had contralateral breast cancer (N = 4), malignant melanoma (N = 2), cancer of the uterine cervix (N = 1), or thyroid cancer (N = 1). The three most common autoimmune diseases were Hashimoto’s thyroiditis (N = 4), rheumatoid arthritis (N = 4), and bronchial asthma (N = 2). Significant cardiovascular disease included history of thromboembolic complications (N = 3), cardiac disease (N = 2), and aneurysm rupture (N = 1).

To identify a potential impact of carboplatin in addition to the treatment with taxanes, an additional analysis was performed comparing taxane-based regimens including carboplatin (EC + PAC/Carbo, EC + PAC/Carbo + Pembro, or TCbHP) and taxane-based regimens not including carboplatin (EC + PAC, APT, or ETC).

In addition, moderate-to-severe PNP, mild PNP, and no PNP were compared with respect to the problems of the NCCN Distress Thermometer indicated by the patients to be associated with their distress. For each comparison, the percentage of patients indicating the corresponding problem was used.

### Statistical Analyses

For the analyses regarding potential associations between PNP or another investigated characteristic and distress, the mean distress score plus standard deviations (SD) and the median distress score plus interquartile ranges (Q1–Q3) were calculated. Univariable analyses were performed with the Kruskal–Wallis test (more than two categories) or the two-sided Wilcoxon Mann–Whitney test (two categories). In addition to the univariable analyses, the factors achieving a *p*-value ≤0.20 in these analyses were subjected to analysis of variance (ANOVA) to further assess their multivariable relevance. Based on the ANOVA model, least-square means were defined as the group means after having controlled for the other model parameters, and associated 95% confidence intervals were derived.

For the comparison of moderate-to-severe PNP, mild PNP, and no PNP with respect to the problems of the NCCN Distress Thermometer, the exact Cochran–Armitage test was used for each problem to test whether there is a trend between the severity of PNP and the number of indications. Due to the number of statistical tests, a multiple testing approach was implemented by means of the false discovery rate (FDR). The FDR is a statistical method used to correct for multiple comparisons. It is a less conservative approach compared to traditional methods, such as the Bonferroni correction. FDR adjusts for the distribution of *p*-values in the data and balances the trade-off between Type II and Type I errors. Specifically, it estimates the proportion of declared positive results that are actually false positives. Thus, an FDR value of 0.05 means that 5% of the declared positive results are truly negative.

For all statistical analyses, the SAS 9.4 software was used (SAS Institute Inc., Cary, NC, USA). In all analyses, *p*-values <0.05 were considered to indicate significance, and *p*-values < 0.10 indicated a trend.

## 3. Results

Distress scores (higher scores representing higher levels of distress) were 6.17 (SD ± 2.41) in patients with moderate-to-severe PNP, 4.21 (SD ± 2.54) in patients with mild PNP, and 4.04 (SD ± 2.24) in patients without PNP, respectively. On univariable analyses, higher distress scores were significantly associated with moderate-to-severe PNP (vs. mild or no PNP, *p* < 0.001; vs. mild PNP vs. no PNP, *p* = 0.003), Karnofsky performance score ≤ 80 (*p* = 0.001), history of autoimmune disease (*p* = 0.035), and hypertension (*p* = 0.002). Trends were found for age ≥65 years (*p* = 0.056), type of chemotherapy (*p* = 0.078), and beta-blocker medication (*p* = 0.072). The analysis investigating the role of carboplatin in addition to the treatment with taxanes did not reveal a significant impact of carboplatin (*p* = 0.374). The mean distress scores and standard deviations with vs. without carboplatin were similar, and the median distress scores were identical. The results of the univariable analyses of all investigated characteristics, including mean distress scores plus standard deviations (SD) and median distress scores plus interquartile ranges (Q1–Q3), are shown in Table 3.

In the subsequent multivariable analyses, moderate-to-severe PNP vs. mild or no PNP (*p* = 0.036), Karnofsky performance score ≤ 80 (*p* = 0.013), and hypertension (*p* = 0.045) were significant. In addition, a trend was found for moderate-to-severe PNP vs. mild PNP vs. no PNP (*p* = 0.099). The complete results of the multivariable analyses are given in Table 4 and Table 5.

In the additional analyses comparing moderate-to-severe PNP, mild PNP, and no PNP with respect to the problems of the NCCN Distress Thermometer indicated by the patients, significant differences were identified after correction for multiple comparisons for nine problems. These problems included worry (*p* = 0.033), loss of interest in usual activities (*p* = 0.033), changes in urination (*p* = 0.033), eating (*p* = 0.033), fatigue (*p* = 0.012), getting around (*p* = 0.012), indigestion (*p* = 0.025), memory/concentration (*p* = 0.022), and tingling in hands/feet (*p* < 0.001). In addition, transportation was almost significant (*p* = 0.050). The results of the whole additional analysis are summarized in Table 6. No patient indicated spiritual/religious concerns as a problem, and substance use was not included in the German version used for this study.

## 4. Discussion

Chemotherapy-induced PNP is not uncommon in breast cancer patients and can significantly impair their quality of life during and following the period of chemotherapy [12,13,14,15,16,17,18,19]. Only very few studies specifically investigated the impact of PNP on the level of distress in these patients [20]. For the design, we used instruments to assess the quality of life, and the endpoints of the corresponding studies varied [8,9,11,20,21,22,23,24,25,26]. No study so far has investigated the effect of PNP on the patients’ distress using the NCCN Distress Thermometer [27,28,29]. Therefore, the present study was performed. It compared moderate-to-severe PNP, mild PNP, and no PNP with respect to mean and median distress scores in patients who were assigned to adjuvant radiotherapy for non-metastatic and received neoadjuvant or adjuvant taxane-based chemotherapy before. In addition, the three PNP groups were compared with respect to the frequencies of the problems indicated by the patients to be relevant for their distress.

According to the results of this study, the severity of chemotherapy-induced PNP was an independent risk factor for distress in patients assigned to adjuvant radiotherapy for breast cancer. Patients with moderate-to-severe PNP had significantly higher mean and median distress scores when compared to patients with mild or no PNP. Significant differences regarding the frequency of the indication by the patients to be relevant for their distress were found for nine problems, including worry, loss of interest in usual activities, changes in urination, eating, fatigue, getting around, indigestion, memory/concentration, and tingling in hands/feet. The difference regarding transportation almost achieved significance. The greatest difference was found for tingling in hands/feet. This finding was not unexpected, since tingling is a typical symptom of PNP [5]. More pronounced symptoms of PNP such as numbness, pain, and tingling in their hands and feet will likely have a negative impact on the patient’s mobility (getting around and transportation) and fine motor skills, which may cause problems with eating. In addition, PNP will likely affect the patient’s psychological well-being including being worried and losing interest in usual activities. Moreover, a potential negative impact of symptoms of PNP on the patient’s general condition may be associated with fatigue. In a recent review article, diabetic PNP was mentioned to cause problems with the urinary tract (similar to the term “changes in urination”), which may also hold true for chemotherapy-induced PNP [38]. Finally, two studies published in 2022 found associations between PNP and cognitive impairment in an elderly population and in patients with human immunodeficiency virus, respectively [39,40]. Moreover, a small prospective study showed that breast cancer patients treated with taxane-based chemotherapy have an increased risk for both PNP and cognitive impairment [41].

In addition to moderate-to-severe PNP, higher distress scores were significantly associated with a worse performance status (Karnofsky performance score ≤ 80) and history of hypertension. The fact that cancer patients with a poor performance status are more distressed than patients in better general conditions was already reported more than 30 years ago [42]. The fact that living with hypertension causes distress was also previously described [43,44]. The agreement between the results of previous studies and the findings of our current study supports the consistency of our data.

However, during the interpretation of our study, its limitations need to be considered, particularly its retrospective design including the risk of hidden selection biases. Another limitation of our study is the fact that education, socio-economic status, and employment could not be investigated but could have an influence on a patient’s level of distress. The same applies to the primary tumor stage and nodal stage. Moreover, the diagnosis of PNP was based on symptoms stated by the patients and physical examination. Additional diagnostic procedures to identify PNP such as electromyography, nerve conduction studies, and neuromuscular ultrasound were not performed [45,46,47]. Finally, since this is a single-county study with a relatively small sample size, the generalizability of our results may be limited.

## 5. Conclusions

The severity of chemotherapy-induced PNP was found to be independently associated with distress in patients assigned to adjuvant radiotherapy for breast cancer. Patients with moderate-to-severe chemotherapy-induced PNP experienced a significantly higher level of distress when compared to patients with mild or no PNP. Treating physicians including radiation oncologists should be aware of this complication and initiate supporting measures as soon as possible. Moreover, patients with moderate-to-severe PNP would likely benefit from personalized monitoring during and following their treatment for breast cancer and should be offered early psychological support. Psychological interventions may include coping skills training, problem-solving training, psychotherapy, social support, acceptance- and commitment-oriented approaches, mindfulness-based stress reduction, and mindfulness-based cognitive therapy. Since our study is retrospective in nature, its results should be validated in prospective trials. These trials should be performed in a larger cohort of patients from different countries to increase the generalizability of the results. Moreover, diagnosis of PNP should include electrodiagnostic testing.

## Figures and Tables

**Table 1:** Chemotherapy regimens used in this study.

Abbreviation	Chemotherapy Regimen
EC + PAC	4 × epirubicin 90 mg/m^2^ + cyclophosphamide 600 mg/m^2^ q2w, followed by 12 × paclitaxel 80 mg/m^2^ q1w
EC + PAC/Carbo	4 × epirubicin 90 mg/m^2^ + cyclophosphamide 600 mg/m^2^ q2w, followed by 12 × nab-paclitaxel 125 mg/m^2^ + carboplatin AUC 1.5 q1w 12 × nab-paclitaxel 125 mg/m^2^ + carboplatin AUC 1.5 q1w, followed by 4 × epirubicin 90 mg/m^2^ + cyclophosphamide 600 mg/m^2^ q2w 4 × epirubicin 90 mg/m^2^ + cyclophosphamide 600 mg/m^2^ q2w, followed by 12 × nab-paclitaxel 125 mg/m^2^ + carboplatin AUC 1.5 q1w + trastuzumab + pertuzumab q3w
EC + PAC/Carbo + Pembro	12 × nab-paclitaxel 125 mg/m^2^ + carboplatin AUC 1.5 q1w, followed by 4 × epirubicin 90 mg/m^2^ + cyclophosphamide 600 mg/m^2^ q2w; combined with pembrolizumab 200 mg 3w 4 × epirubicin 90 mg/m^2^ + cyclophosphamide 600 mg/m^2^ q2w, followed by 12 × nab-paclitaxel 125 mg/m^2^ + carboplatin AUC 1.5 q1w; combined with pembrolizumab 200 mg 3w
APT	12 × paclitaxel 80 mg/m^2^ + trastuzumab
ETC	3 × epirubicin 150 mg/m^2^ followed by 3x paclitaxel 225 mg/m^2^, followed by 3 × cyclophosphamide 2000 mg/m^2^ q2w
TCbHP	6 × docetaxel 75 mg/m^2^ + carboplatin AUC 6 + trastuzumab + pertuzumab q3w 6 × docetaxel 75 mg/m^2^ + carboplatin AUC 6 + trastuzumab q3w

**Table 2:** Distributions of the characteristics investigated for potential associations with the distress score.

Characteristic	*n* Patients	(%)
Peripheral sensory neuropathy		
No	37	37.8
Mild	35	35.7
Moderate to severe	26	26.5
Time of chemotherapy		
Neoadjuvant	59	60.2
Adjuvant	39	39.8
Type of surgery		
Breast-conserving surgery	75	76.5
Mastectomy	23	23.5
Age		
≤64 years	81	82.7
≥65 years	17	17.3
Karnofsky performance status		
≤80	25	25.5
90–100	73	74.5
Body mass index		
<25.0 kg/m^2^	41	41.8
25.0–29.9 kg/m^2^	31	31.6
≥30 kg/m^2^	26	26.5
Personal status		
Living with spouse or partner	73	74.5
Single/widow/living alone	25	25.5
History of other malignancy		
No	90	91.8
Yes	8	8.2
Type of chemotherapy		
EC + PAC	32	32.7
EC + PAC/Carbo	14	14.3
EC + PAC/Carbo + Pembro	18	18.4
APT	5	5.1
ETC	8	8.2
TCbHP	21	21.4
Autoimmune disease		
No	80	81.6
Yes	18	18.4
Significant cardiovascular disease		
No	92	93.9
Yes	6	6.1
Hypertension		
No	67	68.4
Yes	31	31.6
Diabetes		
No	92	93.9
Yes	6	6.1
History of smoking		
<10 pack years	69	70.4
≥10 pack years	29	29.6
Beta-blocker medication		
No	85	86.7
Yes	13	13.3
Main histology		
No special type alone	85	86.7
Other	13	13.3
Histologic grading		
G1 or G2	34	34.7
G3	64	65.3
Triple-negativity		
No	73	74.5
Yes	25	25.5

Abbreviations used for the types of chemotherapy are explained in Table 1.

**Table 3:** Mean and median distress scores of the investigated characteristics, including peripheral sensory neuropathy (univariable analyses).

Characteristics Compared in the Statistical Analysis	Mean Distress Score (±SD)	Median Distress Score (Q1–Q3)	*p*-Value
Peripheral sensory neuropathy			**0.003**
No	4.04 (2.24)	4.0 (2.0–5.0)	
Mild	4.21 (2.54)	4.0 (2.0–6.0)	
Moderate to severe	6.17 (2.41)	6.25 (5.0–8.0)	
Peripheral sensory neuropathy			**<0.001**
No or mild	4.13 (2.38)	4.0 (2.0–5.75)	
Moderate to severe	6.17 (2.41)	6.25 (5.0–8.0)	
Time of chemotherapy			0.247
Neoadjuvant	5.09 (2.68)	5.0 (3.0–8.0)	
Adjuvant	4.39 (2.43)	5.0 (2.5–6.5)	
Type of surgery			0.608
Breast-conserving surgery	4.57 (2.51)	5.0 (2.5–6.5)	
Mastectomy	5.00 (2.67)	5.0 (3.0–8.0)	
Age			0.056
≤64 years	4.43 (2.41)	5.0 (2.5–6.0)	
≥65 years	5.82 (2.88)	6.0 (4.0–8.0)	
Karnofsky performance status			**0.001**
≤80	6.22 (2.40)	6.0 (4.5–8.0)	
90–100	4.14 (2.38)	4.0 (2.0–5.5)	
Body mass index			0.131
<25.0 kg/m^2^	4.01 (2.18)	3.5 (2.0–5.5)	
25.0–29.9 kg/m^2^	5.08 (2.88)	5.0 (2.5–8.0)	
≥30 kg/m^2^	5.21 (2.51)	5.0 (3.0–7.0)	
Personal status			0.436
Living with spouse or partner	4.56 (2.63)	5.0 (3.0–6.5)	
Single/widow/living alone	4.98 (2.28)	5.0 (3.0–6.0)	
History of other malignancy			0.231
No	4.57 (2.50)	5.0 (2.5–6.5)	
Yes	5.81 (2.87)	5.25 (4.0–8.25)	
Type of chemotherapy			0.078
EC + PAC	4.61 (2.67)	4.5 (2.25–7.25)	
EC + PAC/Carbo	6.04 (2.37)	5.75 (5.0–8.0)	
EC + PAC/Carbo + Pembro	3.83 (2.15)	3.0 (2.0–5.0)	
APT	5.10 (2.88)	6.0 (2.5–6.0)	
ETC	6.06 (1.99)	5.5 (4.75–7.0)	
TCbHP	3.93 (2.51)	4.0 (2.5–5.0)	
Chemotherapy including carboplatin			0.374
No	4.92 (2.59)	5.0 (3.0–7.0)	
Yes	4.45 (2.50)	5.0 (3.0–6.0)	
Autoimmune disease			**0.035**
No	4.39 (2.47)	5.0 (2.5–6.0)	
Yes	5.89 (2.56)	6.0 (4.5–8.0)	
Significant cardiovascular disease			0.324
No	4.61 (2.57)	5.0 (2.5–6.5)	
Yes	5.58 (2.01)	5.5 (4.0–7.0)	
Hypertension			**0.002**
No	4.11 (2.35)	3.5 (2.0–6.0)	
Yes	5.87 (2.55)	5.5 (5.0–8.0)	
Diabetes			0.270
No	4.59 (2.47)	5.0 (2.75–6.25)	
Yes	5.83 (3.54)	6.5 (4.0–9.0)	
History of smoking			0.151
<10 pack years	4.41 (2.55)	5.0 (2.0–6.5)	
≥10 pack years	5.28 (2.47)	5.0 (3.5–6.5)	
Beta-blocker medication			0.072
No	4.48 (2.47)	5.0 (2.5–6.0)	
Yes	5.92 (2.75)	6.0 (5.0–8.0)	
Main histology			0.293
No special type alone	4.54 (2.46)	5.0 (3.0–6.0)	
Other	5.50 (3.01)	5.0 (2.5–8.0)	
Histologic grading			0.905
G1 or G2	4.74 (2.59)	4.5 (3.0–6.5)	
G3	4.63 (2.53)	5.0 (2.5–6.5)	
Triple-negativity			0.254
No	4.49 (2.60)	5.0 (2.5–6.0)	
Yes	5.18 (2.32)	5.0 (3.0–7.0)	

Abbreviations used for the types of chemotherapy are explained in Table 1. *p*-Values indicating significance are given in bold.

**Table 4:** Multivariable analysis of variance with three categories of peripheral sensory neuropathy (no vs. mild vs. moderate to severe).

Characteristics Compared in the Statistical Analysis	Least-Square Mean	95% Confidence Interval	*p*-Value *
Peripheral sensory neuropathy			0.099
No	5.0	3.74–6.25	
Mild	5.3	3.98–6.55	
Moderate to severe	6.4	5.39–7.47	
Age			0.859
≤64 years	5.5	4.61–6.39	
≥65 years	5.6	4.18–7.08	
Karnofsky performance status			**0.012**
≤80	6.4	5.27–7.51	
90–100	4.7	3.56–5.92	
Body mass index			0.760
<25.0 kg/m^2^	5.6	4.41–6.75	
25.0–29.9 kg/m^2^	5.8	4.66–6.96	
≥30 kg/m^2^	5.3	3.99–6.62	
Type of chemotherapy			0.262
EC + PAC	5.3	4.03–6.59	
EC + PAC/Carbo	6.5	5.07–7.99	
EC + PAC/Carbo + Pembro	4.7	3.31–6.14	
APT	5.1	3.07–7.16	
ETC	6.5	4.68–8.34	
TCbHP	5.2	3.93–6.47	
Autoimmune disease			0.503
No	5.3	4.41–6.28	
Yes	5.8	4.44–7.13	
Hypertension			**0.049**
No	4.9	3.55–6.22	
Yes	6.3	5.26–7.23	
History of smoking			0.185
<10 pack years	5.2	4.24–6.18	
≥10 pack years	5.9	4.72–7.12	
Beta-blocker medication			0.398
No	5.9	5.10–6.80	
Yes	5.2	3.52–6.84	

Abbreviations used for the types of chemotherapy are explained in Table 1. * The *p*-values were derived from F-tests (type III-SS) in multivariable analyses of variance. *p*-Values indicating significance are given in bold.

**Table 5:** Multivariable analysis of variance with two categories of peripheral sensory neuropathy (no or mild vs. moderate to severe).

Characteristics Compared in the Statistical Analysis	Least-Square Mean	95% Confidence Interval	*p*-Value *
Peripheral sensory neuropathy			**0.036**
No or mild	5.1	3.98–6.27	
Moderate to severe	6.4	5.39–7.46	
Age			0.872
≤64 years	5.7	4.85–6.58	
≥65 years	5.8	4.44–7.23	
Karnofsky performance status			**0.013**
≤80	6.6	5.52–7.65	
90–100	4.9	3.82–6.11	
Body mass index			0.726
<25.0 kg/m^2^	5.8	4.63–6.93	
25.0–29.9 kg/m^2^	6.0	4.92–7.16	
≥30 kg/m^2^	5.5	4.26–6.76	
Type of chemotherapy			0.269
EC + PAC	5.5	4.29–6.76	
EC + PAC/Carbo	6.7	5.33–8.14	
EC + PAC/Carbo + Pembro	4.9	3.60–6.33	
APT	5.3	3.28–7.32	
ETC	6.7	4.92–8.52	
TCbHP	5.4	4.13–6.68	
Autoimmune disease			0.505
No	5.6	4.65–6.46	
Yes	6.0	4.70–7.29	
Hypertension			**0.045**
No	5.1	3.81–6.36	
Yes	6.4	5.50–7.43	
History of smoking			0.174
<10 pack years	5.4	4.48–6.35	
≥10 pack years	6.1	4.99–7.29	
Beta-blocker medication			0.405
No	6.2	5.29–7.02	
Yes	5.4	3.81–6.99	

Abbreviations used for the types of chemotherapy are explained in Table 1. * The *p*-values were derived from F-tests (type III-SS) in multivariable analyses of variance. *p*-Values indicating significance are given in bold.

**Table 6:** Comparison of no peripheral sensory neuropathy (PNP), mild PNP, and moderate-to-severe PNP with respect to the problems indicated by the patients to be associated with their distress.

Problem	No *n* (%)	Mild *n* (%)	Moderate to Severe *n* (%)	FDR *p*-Value
Child care	1 (2.7)	0 (0.0)	2 (7.7)	0.305
Housing	3 (8.1)	1 (2.9)	1 (3.8)	0.319
Insurance	1 (2.7)	2 (5.7)	3 (11.5)	0.218
Financial	2 (5.4)	6 (17.1)	6 (23.1)	0.106
Transportation	0 (0.0)	2 (5.7)	4 (15.4)	0.050
Work/school	0 (0.0)	0 (0.0)	2 (7.7)	0.132
Dealing with children	1 (2.7)	1 (2.9)	2 (7.7)	0.305
Dealing with partner	1 (2.7)	2 (5.7)	2 (7.7)	0.305
Family health issues	0 (0.0)	3 (8.6)	3 (11.5)	0.132
Depression	3 (8.1)	3 (8.6)	6 (23.1)	0.132
Fears	15 (40.5)	13 (37.1)	13 (50.0)	0.319
Nervousness	12 (32.4)	4 (11.6)	6 (23.1)	0.262
Sadness	10 (27.0)	10 (28.6)	10 (38.5)	0.282
Worry	13 (35.1)	14 (40.0)	18 (69.2)	**0.033**
Loss of interest in usual activities	3 (8.1)	9 (25.7)	9 (34.6)	**0.033**
Appearance	5 (13.5)	8 (22.9)	6 (23.1)	0.281
Bathing/dressing	1 (2.7)	3 (8.6)	4 (15.4)	0.132
Breathing	2 (5.4)	4 (11.4)	5 (19.2)	0.132
Changes in urination	0 (0.0)	3 (8.6)	5 (19.2)	**0.033**
Constipation	4 (10.8)	5 (14.3)	3 (11.5)	0.534
Diarrhea	3 (8.1)	6 (17.1)	6 (23.1)	0.132
Eating	5 (13.5)	7 (20.0)	11 (42.3)	**0.033**
Fatigue	14 (37.8)	15 (42.9)	21 (80.8)	**0.012**
Feeling swollen	8 (21.6)	7 (20.0)	9 (34.6)	0.262
Fevers	1 (2.7)	0 (0.0)	2 (7.7)	0.305
Getting around	11 (29.7)	11 (31.4)	19 (73.1)	**0.012**
Indigestion	4 (10.8)	9 (25.7)	11 (42.3)	**0.025**
Memory/concentration	10 (27.0)	12 (34.3)	17 (65.4)	**0.022**
Mouth sores	4 (10.8)	2 (5.7)	6 (23.1)	0.218
Nausea	5 (13.5)	2 (5.7)	8 (30.8)	0.132
Nose dry/congested	13 (35.1)	9 (25.7)	12 (46.2)	0.305
Pain	13 (35.1)	12 (34.3)	15 (57.7)	0.132
Sexual	3 (8.1)	2 (5.7)	5 (19.2)	0.218
Skin dry/itchy	11 (29.7)	12 (34.3)	13 (50.0)	0.132
Sleep	17 (45.9)	18 (51.4)	15 (57.7)	0.282
Tingling in hands/feet	0 (0.0)	23 (65.7)	25 (96.2)	**<0.001**

FDR: false discovery rate (statistical method to correct for multiple comparisons). *p*-Values indicating significance are given in bold.

## Data Availability

The data cannot be shared due to data protection regulations. Only evaluation of anonymized data is allowed according to the responsible ethics committee.
